# Foreign Body Aspiration in Adults (Two Unusual Foreign Bodies; Knife and Tube Tracheostomy)

**Published:** 2017-01

**Authors:** Seyed Mozafar Hashemi, Mohsen Kolahdouzan, Shahab Shahabi, Hamid Talebzadeh, Mohammad Taghi Rezaei

**Affiliations:** 1 *Department of Thoracic Surgeon, Al-Zahra Hospital, Isfahan University of Medical Sciences, Isfahan, Iran.*; 2 *Department of General Surgery, Al-Zahra Hospital, Isfahan University of Medical Sciences, Isfahan, Iran.*

**Keywords:** Asymptomatic, Aspiration, Adult, knife

## Abstract

**Introduction:**

Foreign body aspiration is usually a serious condition that is most common among the pediatric population, and rare in adults. In adults, aspiration may be tolerated for a long time.

**Case Reports:**

Our first case is a 38-year-old man who presented with a 2-day history of swallowing a foreign body. He was completely asymptomatic. Chest X-ray revealed the presence of 5-cm foreign object in the right main bronchus. Rigid bronchoscopy was performed and a knife was removed from the right main bronchus. Second, a 57-year old man with a known case of laryngeal cancer from 15 years previously was admitted for respiratory distress. He had previously undergone a permanent tracheostomy and had received radiotherapy for his cancer. At the first visit, the patient had prominent distress and was transferred to the operating room as an emergency. A tube was seen on chest X-ray. On bronchoscopy, we found the tracheostomy situated in the carina. The cleaved tracheostomy was removed using the grasper, by grasping the cuff line.

**Conclusion:**

We conclude that foreign body aspiration might be completely asymptomatic, especially in an adult. A good history and imaging findings can help us to diagnose and treat the condition carefully.

## Introduction

Foreign body aspiration is usually a serious condition that is most common in the pediatric population (1), but uncommon in adults (2). The etiology of foreign body aspiration is often peanuts, which may be radiopaque on X-ray (3). The majority of patients present with acute symptoms, for example cough, dyspnea and hemoptysis, while others present with chronic symptoms (2,4). Aspiration may be tolerated in adults for a long period of time. Most patients have an underlying disease, such as mental retardation, abnormal swallowing reflex, or neurological problems, for example (5). Diagnosis must be established by means of symptoms, radiography and bronchoscopy. Treatment is removal of the foreign body from the airway tract (2).

***Case 1:***

**Fig 1:**
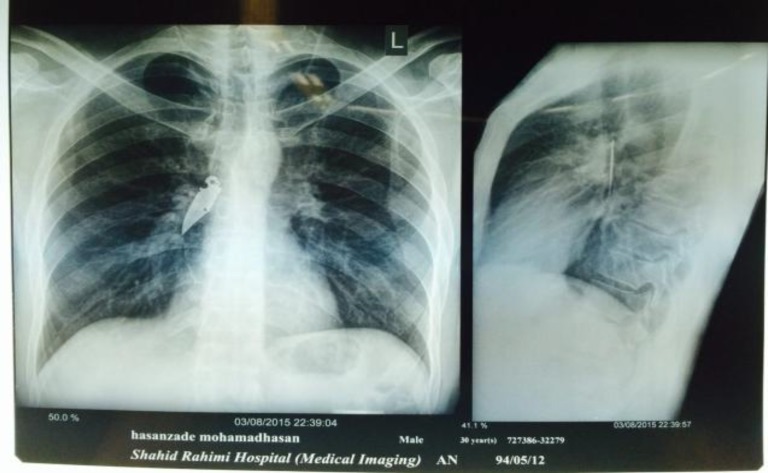
Foreign body in the right main bronchus

A 38-year-old man presented with a 2-day history of swallowing a foreign body. He had been playing with a knife, which was accidentally aspirated after breaking the handle. He has no history of psychiatric problems without any hospitalization or visit a psychiatrist as he said. On presentation to the emergency department, he was asymptomatic. He did not complain of cough, hemoptysis, or dyspnea, and there were no specific findings on physical examination. According to the patient history, a first endoscopy was performed by a gastroenterologist who was not able to find anything. A surgery consultation was then requested. Laboratory findings were within the normal limits. Postero-anterior and lateral chest X-rays revealed the presence of a 5-cm foreign object in the right main bronchus (Fig.1).

The patient’s family were contacted for consent with respect to a possible tracheostomy. 

**Fig 2:**
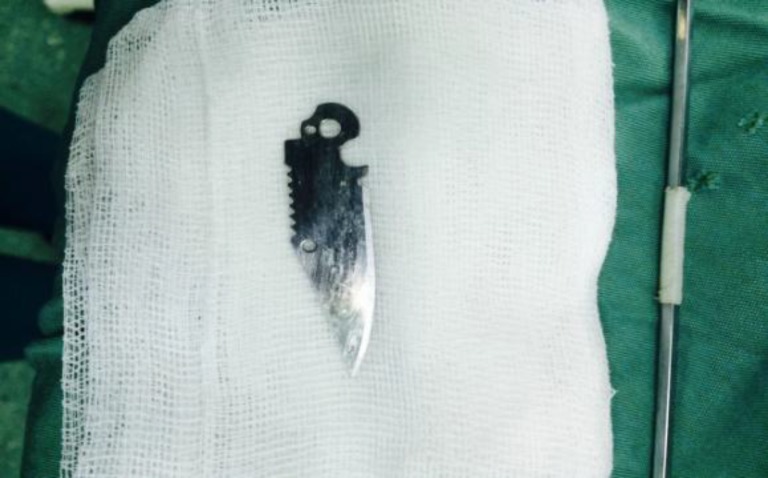
Foreign body removed by rigid bronchoscope

In the operating room, a rigid bronchoscopy was performed under general anesthesia. The 5-cm blade was extracted with a grasper through a bronchoscope from the right main bronchus without complication (Fig.2).

***Case 2:***

**Fig 3:**
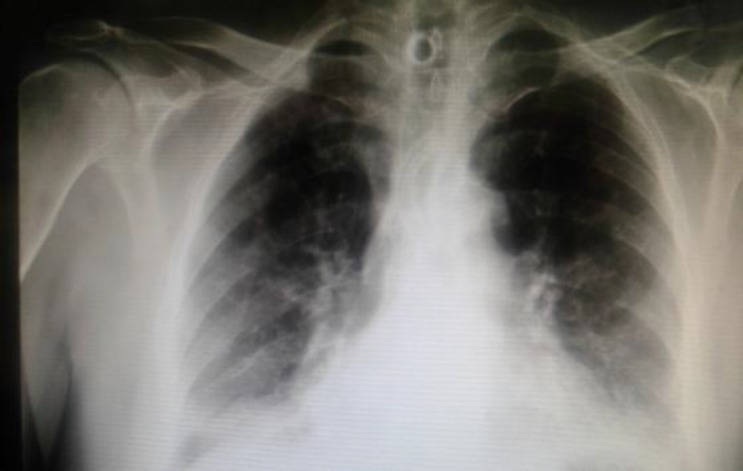
Foreign body visible in the carina

A 57-year old Persian man, with a known case of laryngeal cancer from 15 years previously, was admitted to an emergency ward with a principal complaint of respiratory distress. The patient had previously undergone a permanent tracheostomy and received radiotherapy for his cancer. Three months earlier, the patient visited his physician because of dyspnea. On investigation, stenosis of the tracheostomy site was diagnosed and the permanent tracheostomy was excited and a transient tracheostomy inserted in the patient’s trachea. The patient was also evaluated for cancer recurrence. The patient suctioned his own trachea with an unconventional instrument. On the final time of suctioning, the tracheostomy was cleaved and dropped into the trachea, causing respiratory distress. At the first visit, the patient had prominent distress and was transferred to the operating room as an emergency. On chest X-ray we were able to see the tube (Fig.3). 

**Fig4:**
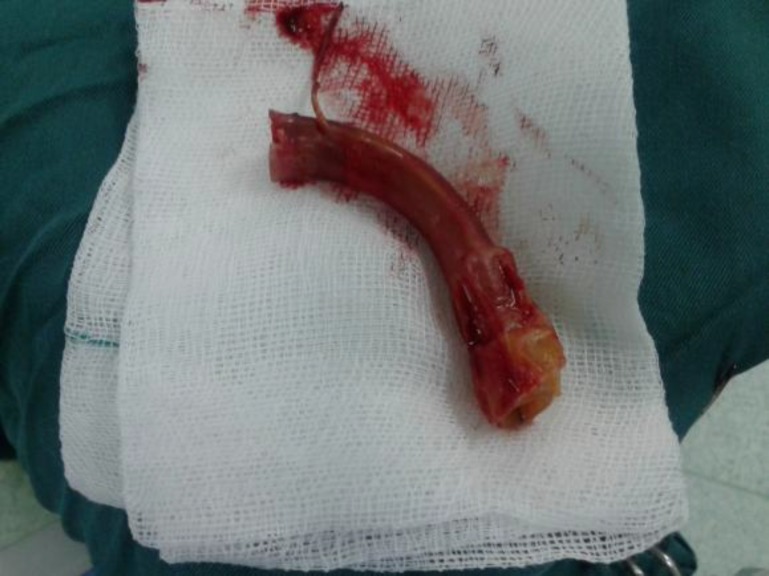
Foreign body removed by bronchoscopy

Initially, we tried to remove the cleaved tracheostomy with a rigid bronchoscope, but the bronchoscope was not able to pass from the tracheostomy orifice because of limitations of the vertebral column motion and tissue disorganization in the context of radiation. Therefore, we tried the procedure a second time by fiber optic bronchoscopy. On bronchoscopy we found the tracheostomy in the carina. A grasper was inserted into the trachea beside the bronchoscope, and the cleaved tracheostomy was removed using a grasper, by grasping of the cuff line (Fig.4).

## Discussion

Foreign body aspiration refers to the condition in which a liquid or solid object blocks the respiratory tract. The right main bronchus is more often involved, similar to our first case. This situation is more common in children, but rarely occurs in adults. Swallowing and cough reflexes are two important mechanisms to prevent this problem. Risk factors such as alcohol use, intubation, neuromuscular disorders, and psychiatric problems, for example, contribute to the problem of foreign body aspiration. Our patients had no such risk factors and no history of addiction or alcohol consumption (4,5).

Unlike children, presentation in adults can range from asymptomatic (as in our first case) to pneumonia, and may be tolerated as a non-specific respiratory problem, such as chronic cough, hemoptysis, dyspnea, or fever (2,4). 

In our first case, the patient was completely asymptomatic, despite the large object and the passing of 3 days. In the second case, the patient was symptomatic.

Diagnosis of foreign body aspiration is based on radiography, computed tomography (CT) scan and bronchoscopy (5). Because of our first case was asymptomatic and the patient had been swallowing, he underwent esophagoscopy. In the second case, because of the reported symptoms, the patient underwent chest X-ray and a planned bronchoscopy.

In total 6–80% of radiographies have negative findings from all foreign bodies, and only 15.7% are radiopaque, like our second case. Treatment involves removal of the foreign body using a rigid or fiberoptic bronchoscopy with a grasper. Chronic cases may be misdiagnosed as a tumor because of inflammation, and surgery may be performed in these cases (2,3).

## Conclusion

We conclude that foreign body aspiration might be completely asymptomatic, especially in an adult. A good history and imaging findings can help us to diagnose and treat the condition carefully.
